# Phototherapy as an alternative in the treatment of chronic spontaneous urticaria

**DOI:** 10.3389/falgy.2024.1468983

**Published:** 2024-11-21

**Authors:** María Inés Giustozzi, Ana Clara Torre, Carla Ritchie, Claudio Alberto Salvador Parisi

**Affiliations:** ^1^ Department of Dermatology, Hospital Italiano de Buenos Aires, Buenos Aires, Argentina; ^2^ Allergy Unit, Hospital Italiano de Buenos Aires, Buenos Aires, Argentina

**Keywords:** phototherapy, urticaria, chronic spontaneous urticaria, therapy, management

## Abstract

Chronic spontaneous urticaria (CSU) is defined as the occurrence of hives, angioedema, or both, lasting for more than 6 weeks. The treatment is based on the use of antihistamines, omalizumab, and/or cyclosporine following a stepwise algorithm recommended by international guidelines with a high level of evidence. Nevertheless, management can be challenging as some patients do not respond to the suggested drugs or have difficulties accessing them for various reasons. In such cases, phototherapy has been reported as a potential treatment option. The evidence on the effectiveness of phototherapy is limited. Most studies have methodological limitations and involve small numbers of patients. A systematic review and meta-analysis of four studies in 2020 concluded that, despite the limited number of randomized controlled trials and the low level of evidence, considering overall efficacy, risk/benefit balance, and costs, narrow band ultraviolet B therapy (NB-UVB) may be a useful adjunct therapy for CSU. Other studies have suggested that the effectiveness of combined antihistamine and phototherapy appears to be more effective than antihistamine alone, although this is based on very low-quality evidence. Additionally, the risk of recurrence was lower with the combination therapy. The objective of this review was to evaluate the role of phototherapy in the treatment of CSU. While randomized studies with a larger number of participants providing a high level of evidence are still needed, we consider phototherapy to be a valuable tool in specific clinical contexts, such as a bridge to the initiation of other medications or until spontaneous remission of the condition occurs.

## 1 Introduction

Urticaria is a common inflammatory disease of the skin and mucous membranes, characterized by the appearance of hives and/or angioedema (1, 2). It is classified as either acute or chronic, depending on whether the symptoms last for less than or more than 6 weeks. In addition, according to the role of different triggers in its onset, it is categorized as inducible or spontaneous (2, 3). Chronic spontaneous urticaria (CSU) is defined as the appearance of symptoms for more than 6 weeks without an identifiable trigger (4).

Chronic urticaria (CU) mainly affects individuals between 20 and 40 years of age, with an estimated prevalence of 1%–4%. CSU accounts for 60% of all CU cases and is estimated to affect 0.1%–1.6% of the world's population (1, 5).

This condition has a significant impact on the quality of life of the patients and their environment, and imposes high direct and indirect economic healthcare costs worldwide (6). The AWARE study showed a considerable impact of CSU on work in Europe, with even higher absenteeism and overall work impairment in Central and South America (general work impairment 33.9 ± 33.9 vs. 26.5 ± 27.5, *P* < 0.001), which was associated with greater disease activity in this region (7). This highlights the substantial indirect costs arising from this condition. The direct CSU-related costs, including medications, outpatient visits, hospital admissions, and laboratory tests, are also high. In the USA, it is estimated that $244 million is spent annually on CU, related to medication, use of medical resources, and absenteeism from work (8), while a study in Argentina estimated an annual expenditure of approximately $1,000 per patient for direct costs alone (9).

The diagnosis of urticaria is clinical. Hives are well-demarcated, erythematous, erythematous lesions of variable size and shape, associated with pruritus and distinguished by their evanescent nature, with a duration of 30 min–24 h. Angioedema is characterized by deeper edema, affecting the reticular dermis, subcutaneous cellular tissue, or mucous membranes, and is often accompanied by burning, tightness, or pain. Angioedema lesions have a slower resolution than wheals, of up to 72 h (2).

CSU is typically self-limiting, remitting spontaneously, but its course can be prolonged and with a tendency to relapse. The average duration is 3–5 years. Approximately 80% of patients remit within the first 12 months, but up to 14% may have persistent disease beyond 5 years (10). Several factors are associated with a poor prognosis and longer disease duration, including thyroid autoimmunity, concurrent angioedema, insufficient response to a standard dose antihistamine (with 51% persistence at 2 years and 66% at 5 years), onset after age 45, the presence of concomitant induced CU, intolerance to non-steroidal anti-inflammatory drugs, and a recurrent course. Recurrence is defined as the appearance of new lesions 6 months after resolution of symptoms and discontinuation of controller therapy (7, 10).

Treatment of CSU is based on antihistamines, omalizumab, and/or cyclosporine according to the stepwise therapeutic algorithm recommended by international guidelines with a high level of evidence. Despite clear treatment guidelines, management can be challenging in clinical practice due to multiple reasons. A variable percentage of patients does not respond to the suggested drugs or have difficulties accessing them for various reasons (2). In these cases, phototherapy has been reported as a potential treatment option. Despite its proven efficacy in conditions other than CSU and its excellent safety profile, evidence for its usefulness in the treatment of CSU is limited.

The aim of this review was to evaluate the role of phototherapy in the treatment of CSU and its possible indication profile in selected patients.

To perform this review, articles in English were searched in PubMed using the keywords “phototherapy” and “chronic urticaria”. The identified articles were then analyzed and selected by the group of authors.

## 2 Current therapeutic strategies in CSU

Treatment of CSU based on the EAACI/GA^2^LEN/EuroGuiDerm/APAAACI 2021 guideline consists of a stepwise management of CSU with reassessment of therapy every 2–4 weeks or sooner if symptoms are intolerable. It aims for complete symptom control.

Second-generation oral antihistamines at conventional doses are the first-line, with a dose increase of up to 4-fold in the event of insufficient response. This management may not be effective in 40%–45% of patients (2).

When antihistamines fail, treatment with omalizumab, an anti-IgE monoclonal antibody at a dose of 300 mg every 4 weeks, is recommended (2). Omalizumab was approved in 2014 by the US Food and Drug Administration for patients with CSU in the United States and Europe (11, 12). Its efficacy and safety were demonstrated with a high level of evidence in the randomized, double-blind, placebo-controlled phase III trials ASTERIA I and ASTERIA II at different dosages in patients on approved doses of antihistamines (13). Its long-term safety profile is excellent and there is no need to request studies prior to initiation of the drug (12). In case of lack of response to conventional doses, it is suggested to increase the dose or decrease the dosing interval up to a maximum dose of 600 mg every 2 weeks.

In patients who do not show disease control with omalizumab, the use of cyclosporine at a dose of 3.5–5 mg/kg per day is suggested (2, 14). Its efficacy in CSU has been demonstrated in combination with second generation antihistamines compared to a placebo group, in controlled and open trials (2). Nevertheless, its indication is off label in CSU. Adverse effects (nephrotoxicity, hypertension, headache, myalgia, risky drug interactions) and contraindications [systemic malignancy, renal failure, uncontrolled hypertension, psoralen + ultraviolet A (PUVA) phototherapy, uncontrolled infections, and hypersensitivity to cyclosporine] should be considered before prescribing cyclosporine (15). Due to this safety profile, it is only recommended for CSU refractory to the combination of supramaximal doses of antihistamines and omalizumab. Despite these clear guidelines and the high level of evidence supporting them, it is estimated that approximately 30% of patients do not respond to the suggested drugs (2).

Currently, new treatment approaches focus on specific interleukins (ILs), signaling pathways, and mast cell receptors. Several drug studies are ongoing for targets such as FcεRI, C5aR, mas-related g protein-coupled receptor X2 (MRGPRX2), Siglec-8, KIT, and IL-4Rα receptors, as well as Bruton tyrosine kinase (BTK) and spleen tyrosine kinase (SYK) signaling pathways. Other therapeutic targets include the mediators histamine, tryptase, IL-5, IL-17 and IL-31, and anti-IgE signals that activate autoallergens (4). Dupilumab, approved for the treatment of atopic dermatitis since 2017, has been licensed for the use in CSU in adult patients in Japan and is currently under review by the FDA. These drugs could address the multiple contraindications to cyclosporine and provide an option for patients who do not respond to omalizumab, although cost is a potential issue.

Regarding phototherapy, the 2020 evidence-based clinical practice guideline of the Korean Academy of Asthma, Allergy and Clinical Immunology and the Korean Dermatological Association recommends adding NB-UVB phototherapy to antihistamine treatment for adults and children with symptomatic CSU or dermographism who do not respond to antihistamines alone (16). However, the EAACI/GA^2^LEN/EuroGuiDerm/APAAACI guideline argues that only very low-quality evidence is available, and do not recommend this approach (2).

## 3 Special scenarios and constraints in the treatment of CSU

The management of urticaria can be challenging in clinical practice. Beyond the lack of response of some patients to the recommended treatments, other factors may prevent access to these therapies.

Firstly, contraindications may limit the use of certain therapies, such as hypersensitivity reactions to omalizumab or conditions that contraindicate the use of cyclosporine. In addition, some patients may experience adverse effects that require discontinuing previously indicated treatments, such as intolerance to antihistamines, development of serum sickness from omalizumab, or renal failure from cyclosporine. On the other hand, both cyclosporine and omalizumab are expensive drugs for patients in developing countries who have no or partial healthcare coverage and cannot afford the costs (17).

Finally, there are special contexts, such as the COVID-19 pandemic, have highlighted the need for other therapeutic options (3, 18).

## 4 Phototherapy

Phototherapy is an effective therapeutic tool in dermatology that uses ultraviolet light to treat various skin diseases (19). Despite the development of highly effective biological drugs for conditions such as psoriasis or atopic dermatitis, UVA and UVB therapy are still mainstays in their treatment (20). In addition to its proven efficacy, phototherapy is inexpensive and associated with few adverse effects.

UVB phototherapy is phototherapy with a wavelength between 280 and 320 nanometers (nm). It is further divided into broadband UVB therapy (280–320 nm) and narrowband UVB therapy (311 nm). UVA phototherapy has a wavelength between 320 and 400 nm. The depth that the radiation reaches in the skin depends directly on its wavelength; therefore, UVB, having a shorter wavelength, is absorbed by the epidermis and the superficial portion of the dermis, while UVA waves are longer and penetrate the dermis. PUVA phototherapy combines a photosensitizer, psoralen orally or topically, with subsequent UVA irradiation (21, 22).

### 4.1 Mechanisms of action of phototherapy in CSU

The mechanisms of action of phototherapy are explained by a complex interaction of simultaneous effects during UV irradiation (21), making it an effective therapy for managing numerous dermatological conditions. Six key effects of phototherapy have been described: immunomodulatory, proapoptotic, antipruritic, antifibrotic, propigmentary, and pro-prebiotic.

The immunomodulatory effect of phototherapy is exerted through the release of immunomodulatory molecules, regulation of cell migration, and induction of immunosuppression, which restores mechanisms of immune tolerance and suppresses pathogenic inflammation (23). It also decreases natural killer cell activity, lymphocyte proliferation, and the release of proinflammatory cytokines by Th1 lymphocytes (IL2,IFN-γ), and stimulates the production of IL-10, an anti-inflammatory cytokine (17, 24, 25). This mechanism plays an important role in the treatment of psoriasis, atopic dermatitis, scleroderma, and T-cell lymphomas. On the other hand, phototherapy induces apoptosis in various types of cells, including keratinocytes and epidermal T lymphocytes via the activation of Fas ligand and p53, explaining its antiproliferative effect in psoriasis. Its propigmentary effect is characterized by the interruption of the anti-melanocyte cytotoxic response and the repopulation of these cells and their precursors in the interfollicular epidermis by the production of proopiomelanocortin and α-MSH, and the depletion of cutaneous anti-melanocytic CD8T cells, which explains its efficacy in vitiligo treatment (21). Furthermore, induction of collagen-degrading matrix metalloproteinases with antifibrotic mechanisms can be used to treat scleroderma or sclerodermiform graft-vs.-host disease. Multiple factors are involved in the antipruritic effect, such as downregulation of Th2 cytokines including IL-31, degranulation of mast cells, as UV light-based therapies can trigger the migration of mast cells via cis-Urocanic acid and the secretion of PAF because low dosages of UVB increase the threshold of mast cell degranulation, and release of β-endorphins. Finally, phototherapy modifies the skin microbiome by selection of UV-resistant microbial species, decrease of *Staphylococcus aureus*, and increase of immunostimulatory microbial products, which may play a role in the treatment of atopic dermatitis (23).

The pathophysiology of urticaria is not fully understood (2), but mast cells are considered to play a key role. Their activation and degranulation in the skin releases histamine and other mediators [tryptase, prostaglandin D2 (PGD2), TNF, IL-4, IL-5, IL-13, IL-17 and IL-31] leading to sensory nerve activation, vasodilatation, plasma extravasation, and cell recruitment (T-cells, eosinophils, and basophils). The signals that activate the mast cell are heterogeneous and include cytokines and antibodies, autoantibodies against IgE or the high-affinity IgE receptor (FcεRI), and different factors of the extrinsic coagulation cascade (factor Xa and thrombin) (4). In CSU, these activating signals are believed to arise from autoimmune mechanisms. Two types of autoimmunity have been described. Type I autoimmunity (“autoallergic CSU”) is mediated by IgE antibodies against self-antigens, such as anti-IL-24 IgE and anti-TPO IgE, which activate mast cells and/or basophils *in vitro*. Cross-reactivity between proteins, such as thyroid peroxidase (TPO), which is not present in the skin, and eosinophil peroxidase (EPO), which is present in the skin, as well as the expression of self-allergens in the skin, such as IL-24, may explain why the IgE-autoallergen interaction leads to mast cell activation in the skin but not in other organs. Type IIb autoimmunity involves mast cell-activating IgG autoantibodies directed against IgE and FcεRI (2).

Proapoptotic, immunomodulatory, and antipruritic effects of phototherapy may be involved in its mechanism of action on CSU. However, the exact mechanism of action by which this therapy is effective in the treatment of CSU remains unknown, although two hypotheses have been proposed in the literature. First, UV light shows a dual effect on cutaneous mast cells by triggering a mild but significant release of histamine at rest, but suppresses this same release by up to 90% when the cells are appropriately stimulated. This would lead to a decrease in the production and release of histamine and pro-inflammatory cytokines resulting from its degradation. In addition, it would induce apoptosis of dermal mast cells. However, the evidence for this mechanism is controversial (26). Secondly, phototherapy may act on the pathophysiology of urticaria through its systemic immunoregulatory effect. As the involvement of T-lymphocytes in the pathophysiology of CSU has been demonstrated, this immunoregulatory role could explain the therapeutic effects of phototherapy in treating this condition (26).

### 4.2 Effectiveness of phototherapy in CSU

In 1983, Midelfart et al. reported the first case of improvement of CU after PUVA treatment in a patient with concomitant vitiligo and CU (17). Subsequently, promising results have been reported in the literature for NB-UVB, UVA, and PUVA. However, it is striking that since that initial report only case series or studies with a limited number of patients with CSU treated with phototherapy have been published. These studies often have relatively small sample sizes and multiple biases, and those with larger patient numbers are retrospective or include patients who do not have CU refractory to antihistamines (17).

In 1984, Hannuksela et al. described the first series of 15 patients treated with NB-UVB, without clear inclusion criteria or outcome measures (27). Subsequently, Olafsson et al. reported promising results with UVA plus placebo and PUVA in 19 patients in a randomized double-blind study. They found no difference between these two phototherapy modalities (28).

A 2008 randomized controlled clinical trial compared patients with CSU treated with combined NB-UVB therapy plus antihistamines with a control group receiving antihistamine monotherapy. The former group showed a statistically significant difference in the reduction of the Weekly Urticaria Activity Score (UAS7). After 10 sessions, there was a mean UAS7 reduction of 16.7 points compared to 2.24 in the control group. Furthermore, during the 90-day follow-up, a shorter remission period was observed in patients treated with antihistamine monotherapy. The authors suggest that this long-term improvement in the NB-UVB group may be related to a long-lasting immunoregulatory effect (29).

In an open-label study, Aydogan et al. observed improvement in the Outcome Scoring Scale (OSS) when phototherapy was administered to 22 patients with no response to antihistamines. No patients were included as a control group. Complete improvement, marked improvement, and moderate improvement were reported in 45%, 31%, and 22% of patients, respectively (30). Currently, this clinimetric measure is not used for urticaria studies; however, it makes this study comparable to a previous retrospective study from 2004 where it was also used. In the latter, which included 94 patients, the authors observed 40% disease resolution, 15% marked improvement, and 45% moderate improvement at the end of treatment with NB-UVB, 3–4 times a week for 30–45 sessions (17, 31).

Khafagy et al. found that NB-UVB and PUVA were equally effective in 24 patients who were non-responders to antihistamines. Overall, 58.3% of patients improved in the NB-UVB and 50% in the PUVA group, with a difference that was not statistically significant (*p* > 0.05) (26).

In contrast, Bishnoi et al. demonstrated superiority of NB-UVB over PUVA. They conducted a prospective, randomized, observer-blinded study comparing the efficacy of NB-UVB (group A) and PUVA (group B) in 50 patients with steroid-dependent CU unresponsive to antihistamines at supramaximal doses. Three sessions per week were performed for 90 days and patients were followed for a further 90 days. Treatment efficacy was measured using the UAS7, with a mean decrease of 61.4% in Group A and of 70.9% in Group B at the end of treatment. NB-UVB phototherapy was shown to be statistically more effective (*p* = 0.001). The limitation of this study was the absence of a control group that did not receive phototherapy, making it impossible to evaluate whether the results are due to the characteristic spontaneous remission of the condition. While we can confirm that NB-UVB phototherapy was more effective than PUVA, we cannot conclude it was superior to antihistamines when used as monotherapy. This study also evaluated the effect of phototherapy on serum IgE levels, autologous serum skin tests, and autologous plasma skin tests. A statistically significant decrease in serum IgE levels was evident only in patients treated with PUVA, while a significant reduction in skin test positivity was observed in both groups. The possible mechanism behind this reduction is the systemic immunomodulation generated by this treatment. In addition, this study assessed other causes, beyond the disease activity itself, that may influence the response to treatment. Of the 54% of patients with persistent mild pruritus, 20% were found to have hypothyroidism, more than 50% had phototherapy-induced xerosis, and 32% had atopic dermatitis. All responded well to the application of emollients. This raises questions about the usefulness of phototherapy in patients presenting with concomitant urticaria and atopic dermatitis (17).

The most recent clinical study, published by Sheikh et al. in 2019, enrolled 80 patients with CSU aged between 13 and 62 years. Half of them were treated with NB-UVB phototherapy and desloratadine, and the other half with antihistamines alone. Two weekly sessions of phototherapy were performed and results were measured at 8 and 16 weeks after treatment and 4 weeks after discontinuation of treatment. Comparison of the two groups showed a statistically significant reduction (*p* < 0.01) in UAS7 in the intervention group compared to the control group receiving antihistamine monotherapy alone. At week 8, the mean UAS7 in the first group was 12.03 compared to 21.43 in the second group, and at week 16 the mean UAS7 had decreased to 3.54 and 17.16, respectively. Notably, one month after discontinuing treatment, the UAS7 score remained low in patients in the phototherapy group, while it increased in patients in the loratadine-only group. This suggests that NB-UVB phototherapy when combined with antihistamines results in longer periods of disease remission (25).

In 2020, a systematic review and meta-analysis of three studies, which did not include the above article, concluded that, despite the limited number of randomized controlled trials and the low level of evidence, based on overall efficacy, risk-benefit balance, and costs, NB-UVB may be a useful therapeutic adjunct for CSU (24).

In a similar study, Chen et al. included a total of 713 participants from nine randomized controlled trials published between 2008 and 2020, making it the largest to date. Seven studies had not been included in previous reviews as they were written in Chinese. All the studies compared antihistamines as monotherapy with combination therapy involving phototherapy. The findings suggested that the efficacy of the combination of antihistamines with phototherapy was more effective than antihistamines alone, although with a low level of evidence. In addition, the risk of recurrence was significantly lower with combination therapy (*p* < 0.00001). Despite these promising results, important limitations were found: most participants were from China, introducing geographical bias, only one study reported observer blinding, outcome measurement was variable between studies, and different regimens of NB-UVB phototherapy were used (19). For example, in the study by Berroeta et al. the median number of phototherapy treatment sessions for CU patients was 22, Ening et al. administered 20 sessions, and Sheik et al. performed a total of 16 sessions over an 8-week period (25).

Only one study measured the speed of response, a statistically significant decrease in UAS7 by day 15 of treatment, around the seventh session (17).

### 4.3 Reported adverse effects

Adverse effects of phototherapy may be short or long term. Erythema is the most common acute effect, with UVB-related erythema occurring after the first 24 h after exposure, while erythema caused by PUVA is more delayed and develops between 48 and 72 h later. Pruritus is another acute side effect, which may result from skin xerosis, improving with emollients, or may have an idiopathic cause. For PUVA phototherapy, gastrointestinal intolerance to psoralen and occasionally dizziness or headache may occur. In addition, due to photosensitization of the cornea and retina, assessing the risk of cataract development and indicating ocular protection is important (22).

On the other hand, the chronic consequences of cumulative high doses of UV radiation have been studied, as they lead to photo ageing and an increased risk of skin cancer. A significantly increased risk of developing squamous cell carcinoma has been demonstrated after more than 150 PUVA treatments, and a much higher risk was reported after 350 PUVA treatments (21). Data from trials evaluating the effect of NB-UVB therapy on increased skin cancer risk are inconclusive.

The adverse effects reported for the use of phototherapy in CSU are similar to those described in the literature for other conditions. Khafagy et al. reported erythema in 25% of patients treated with NB-UVB and in 16.7% of those treated with PUVA. As expected, patients treated with PUVA showed a significantly higher rate of gastrointestinal discomfort than those treated with NB-UVB (26). Another study reported nausea in the PUVA-treated group, and in both the PUVA and NB-UVB groups, xerosis, tanning, and melasma were observed, with no statistically significant difference (17). Ening et al. reported a 9% erythema and pruritus rate secondary to NB-UVB therapy.

Regarding the risk of carcinogenesis, all the studies presented indicated less than 30 sessions, a number far below the recommended limit (21).

Although phototherapy is considered a safe therapy in children and is mainly used from an early age in atopic dermatitis, only three studies included pediatric patients for the treatment of CSU. The youngest patient with CSU reported in these studies was 12 years old (Table 1). However, the number of patients under 18 years is not specified, and there is no reference to specific adverse effects in this specific group.

**Table 1:** Studies and case reports of CU treated with phototherapy in the English language.

Study	Subject	Number of cases	Age	Sex	Light source	Frequency	Number of sessions	Phototherapy dosage	Follow up	Study design	Efficacy	Evidence level*
Midelfart et al., (32)	CSU	1	31 years	Female	PUVA	Four times a week during 6 months, subsequently once a week during 1 month	120	Total dose 700 J/cm^2^	10 months	Case report	Partial improvement of symptoms	3
Hannuksela et al., (27)	Chronic inducible urticaria (CIU)	15 (6 p. symptomatic dermographism, 4 p. cholinergic urticaria, 2 p. cold urticaria, and 3 p. non-specific CU)	14- 62 years; Mean age 31.5 years	Male 4; Female 11	BB-UVB	Three times a week during 1 month, subsequently once a week during 2 months	Mean 22 (13–27)	Initial dose 0.02 J/cm^2^ and was gradually increased to a mean of 0.4 (range 0.08–0.7) J/cm^2^. Median top dose 0.4 J/cm^2^ (0.08–0.7 J/cm^2^)	2 months	Data not available	Excellent response: 47% (7 p.) Clearly better: 26% (4 p.) No or slight response: 26% (4 p.)	3
Olafsson et al., (28)	CSU	19	25 - 73 years Mean age 48.3 years	Data not available	PUVA (G1**, 11 p.) UVA plus placebo (G2**, 8 p.) UVA plus placebo	Two times a week during 2 months	Data not available	Initial dose not reported. The doses were gradually increased 1 J/cm^2^. The maximum dose was 15 J/cm^2^.	3 months	Data not available	G1 Improvement: 7 p. No change: 3 p. Worse: 1 p. G2 Improvement: 5 p. No change: 3 p.	3
Berroeta et al., (31)	CU	94 (88 CSU, 6 CIU)	12 -81 years Mean age 44 years	Male 26; Female 68	NB-UVB	Three times a week	Mean 22 (2–37)	Initial dose 70% of MED. Incremental regimen such as 10% every third treatment. Median top dose 1,238 mJ/cm^2^ (100–2,111 J/cm^2^)	Data not available	Retrospective review	Clearance: 40% Marked improvement: 15% Moderate improvement: 45% Unsuccessful:28%	3
Engin et al., (29)	CSU	81	18–62 years. Mean age 34 years	G1 Male 11; Female 34 G2 Male 14; Female 19	NB-UVB	Three times a week during 1.5 months	20	Initial dose: 200 J cm^2^ Incremental regimen 10%–20%. Median top dose: 1.2 J/cm^2^ (8–13 J/cm^2^)	3 months follow-up after the treatment	Randomized Controlled Trial with 2 groups. G1: NB-UVB+antihistamines, 48 p. G2: Only antihistamines, 33 p.	Mean UAS7 G1 Baseline: 34.22 Session 10: 22.6 Session 20: 17.4 G2 Baseline: 33.42 Session 10: 27.3 Session 20: 20.7	2+
Aydogan et al., (30)	CSU	22	19- 64 years Mean age 39.2 years	Male 3 Female 19	NB-UVB	Three- or four-times a week	Median 31.4 (9- 44)	Initial dose: 50%–70% of MED Incremental regimen 0%–20% Median top dose 9.46 J/cm^2^ (1.1–16.4 J/cm^2^)	6 months to 1 year follow up after treatment	Data not available	Clearance: 10 patients (45%) Marked improvement: 5 patients (22%) Moderate improvement: 7 patients (31%)	3
Khafagy et al., (26)	CSU	24	G1 Mean Age 30.25 (21–43) years G2 Mean Age 35.33 years (14–58)	G1 Male 4; Female 8 G2 Male 3; Female 9	PUVA NB-UVB	Three times a week	Maximum of 20	Initial dose of PUVA: Skin type I 0.5 J/cm^2^ Skin type II 1.0 J/cm^2^ Skin type III 1.5 J/cm^2^ Skin type IV 2 J/cm^2^ Skin type V 2.5 J/cm^2^ Skin type VI 3 J/cm^2^ (15). Increase in the UVA dose of 0.5 J/cm^2^, 1 J/cm^2^ and 1.5 J/cm^2^ for skin types I and II, III and IV, and V and VI, respectively Mean total dose 76.75 J/cm^2^ Initial dose of NB-UVB Skin types I and II 0.3 J/cm^2^ Skin types III and IV 0.5 J/cm^2^ Skin types V and VI0.8 J/cm^2^. Incremental regimen 0%–20% Mean total dose 37.83 J/cm^2^	Data not available	Randomized study with 2 groups. G1: PUVA, 12 p. G2: NB-UVB, 12 p.	Mean UAS7 G1 Before treatment: 15.08 After treatment: 11.50 G2 Before treatment: 15.75 After treatment: 11.00	2+
Bishnoi et al., (17)	Steroid-dependent, antihistamine refractory CSU	50	19–65 years G1 Mean Age 35.5 years G2 Mean Age 33.7 years	Male: female ratio 2:3 in both groups	NB-UVB PUVA	Three to five times a week during 3 months	38–64	Initial dose: 50%–70% of MED. Advance treatment by 10% of MED. Median top dose not reported	3 months of follow-up after treatment	Randomized prospective, observer blinded comparative study, with 2 groups. G1: NB-UVB 25p. G2: PUVA 25 p.	Mean UAS7 G1 Baseline: 5.0 90th day: 1.4 G2 Baseline: 4.9 90th day: 1.9 **OSS** G1 Baseline: 1.3 90th day: 4 G2 Baseline: 1.6 90th day: 3.9	2+
Sheikh et al., (25)	CSU	80	13–62 years G1 Mean Age 30.54 years G2 Mean Age 33.40 years	G1 Male 13; Female 22 G2 Male 10; Female 27	NB-UVB	Two times a week during 2 months	16 sessions	Initial dose: 200 J/cm^2^ Incremental regimen 10%–20%. Maximum dose of 1630 J/cm^2^ Median top dose not reported	1 month of follow-up after treatment	Randomized study with 2 groups. G1: NB-UVB plus antihistamines, 40 p. G2: antihistamines, 40 p.	Mean UAS7 G1 Baseline: 33 4 weeks, 8 sessions: 12.03 8 weeks, 16 sessions: 3.54 G2 Baseline 31.97 4 weeks 21.43 8 weeks 17.26	2+

BB-UVB, broad band ultraviolet B; CU, chronic urticaria; G, group; CSU, chronic spontaneous urticaria; MED, minimal erythema dose; NB-UVB, narrowband ultraviolet B; OSS, outcome scoring scale; PUVA, psoralen + ultraviolet A; UAS7, weekly urticaria activity score; UVA, ultraviolet A; VAS, visual analogue score.

* Evidence level according to SIGN classification.

** Group.

### 4.4 Contraindications in phototherapy

While the safety profile of phototherapy is very good, there are relative and absolute contraindications to its administration, which vary in the literature. Absolute contraindications to targeted phototherapy are diseases associated with increased photosensitivity, such as lupus or dermatomyositis, or an increased risk of skin cancer, including xeroderma pigmentosum or nevoid basal cell carcinoma syndrome, also called Gorlin's syndrome (21). Performing an antinuclear antibody test prior to initiation of therapy is not recommended unless there is a history of photosensitivity. Relative contraindications include a history of melanoma, a history of non-melanoma skin cancer, actinic keratoses, a history of treatment with arsenic or ionizing radiation due to the increased risk of skin cancer, and use of immunosuppressive drugs including cyclosporine A, azathioprine, mycophenolate mofetil, and tacrolimus. While there is no evidence of teratogenicity, PUVA is contraindicated during pregnancy because of the use of psoralen (21). On the positive side, UVB phototherapy, both broadband and narrowband, is considered safe for use during pregnancy and lactation.

## 5 Discussion

There are various clinical scenarios in which the recommendations of the international guidelines for the management of chronic urticaria may be impractical or insufficient for the treatment of patients. Three reasons have been identified for the need to resort to treatments outside the therapeutic algorithm proposed in the guidelines. First, it may be due to a lack of response to standard therapy. Second, it may be required because of lack of accessibility due to high medication costs. Finally, different strategies may be considered based on the safety profile of the available drugs. In certain clinical practice settings, these scenarios are common, as one in four patients do not respond to antihistamines at supramaximal doses and require treatment with omalizumab or cyclosporine (7).

Based on the evidence in the literature reviewed above, combined therapy with phototherapy and antihistamines appears to be a valuable therapeutic option in cases where standard therapy is insuffcient, as it demonstrates superior efficacy in controlling CU compared to antihistamine monotherapy. Furthermore, combination therapy may achieve longer-lasting symptomatic relief (19). Despite these benefits, it is important to acknowledge that phototherapy is available only at a limited number of centers, which poses significant challenges to accessibility. Additionally, the requirement for patients to commit time for weekly treatments may be a barrier to its widespread recommendation.

Although randomized studies with a larger sample sizes providing higher level of evidence regarding the role of phototherapy in general, and NB-UVB phototherapy in particular, in the treatment of CSU are still needed, it may be a valuable tool in specific clinical contexts, as a bridge to the initiation of other medications, or until spontaneous remission of CSU occurs.

## References

[1] Sánchez-Borges M Ansotegui IJ Baiardini I Bernstein J Canonica GW Ebisawa M The challenges of chronic urticaria part 1: epidemiology, immunopathogenesis, comorbidities, quality of life, and management. World Allergy Organ J. (2021) 14:100533. 10.1016/j.waojou.2021.100533 34221215 PMC8233382

[2] Zuberbier T Abdul Latiff AH Abuzakouk M Aquilina S Asero R Baker D The international EAACI/GA^2^LEN/EuroGuiDerm/APAAACI guideline for the definition, classification, diagnosis, and management of urticaria. Allergy. (2022) 77:734–66. 10.1111/all.15090 34536239

[3] La Forgia MP Torre AC Infante L Saravia AE Cannavó A Song A. Urticaria y COVID-19. Dermatol Argent. (2022) 28:30–6. 10.47196/da.v28i1.2248

[4] Kolkhir P Giménez-Arnau AM Kulthanan K Peter J Metz M Maurer M. Urticaria. Nat Rev Dis Primers. (2022) 8:61. 10.1038/s41572-022-00389-z 36109590

[5] Lacour J-P Khemis A Giordano-Labadie F Martin L Staumont-Salle D Hacard F The burden of chronic spontaneous urticaria: unsatisfactory treatment and healthcare resource utilization in France (the ASSURE-CSU study). Eur J Dermatol. (2018) 28:795–802. 10.1684/ejd.2018.3446 30698148

[6] Vena GA Maurer M Cassano N Zuberbier T. Alternative treatments for chronic spontaneous urticaria beyond the guideline algorithm. Curr Opin Allergy Clin Immunol. (2017) 17:278–85. 10.1097/ACI.0000000000000372 28537934

[7] Gonçalo M Gimenéz-Arnau A Al-Ahmad M Ben-Shoshan M Bernstein JA Ensina LF The global burden of chronic urticaria for the patient and society. Br J Dermatol. (2021) 184:226–36. 10.1111/bjd.19561 32956489

[8] Maurer M Raap U Staubach P Richter-Huhn G Bauer A Oppel EM Antihistamine-resistant chronic spontaneous urticaria: 1-year data from the AWARE study. Clin Exp Allergy. (2019) 49:655–62. 10.1111/cea.13309 30415478

[9] Parisi CAS Ritchie C Petriz N Morelo Torres C. Direct medical costs of chronic urticaria in a private health organization of Buenos Aires, Argentina. Value Health Reg Issues. (2016) 11:57–9. 10.1016/j.vhri.2016.07.008 27986199

[10] Dabija D Tadi P Danosos GN. Chronic Urticaria. In: StatPearls. Treasure Island (FL): StatPearls Publishing (2023). https://www.ncbi.nlm.nih.gov/pubmed/32310370 32310370

[11] Kumar C Zito PM. Omalizumab. In: StatPearls. Treasure Island (FL): StatPearls Publishing (2023). https://www.ncbi.nlm.nih.gov/pubmed/31424767

[12] Wu KCP Jabbar-Lopez ZK. Omalizumab, an Anti-IgE mAb, receives approval for the treatment of chronic idiopathic/spontaneous urticaria. J Invest Dermatol. (2015) 135:13–5. 10.1038/jid.2014.362 25501377

[13] Cubiró X Spertino J Rozas-Muñoz E Serra-Baldrich E Puig L. The effectiveness of omalizumab treatment in real-life is lower in patients with chronic urticaria longer than 18 months’ evolution and prior immunosuppressive treatment. Actas Dermosifiliogr. (2019) 110:289–96. 10.1016/j.ad.2018.09.009 30360885

[14] Pereyra-Rodriguez JJ Galán Gutiérrez M Armario-Hita JC Ruiz-Villaverde R. Prevalence of chronic urticaria refractory to antihistamines in Andalucia, Spain. Dermatol Ther. (2020) 33:e13866. 10.1111/dth.13866 32558086

[15] Menter A Gelfand JM Connor C Armstrong AW Cordoro KM Davis DMR Joint American academy of dermatology-national psoriasis foundation guidelines of care for the management of psoriasis with systemic nonbiologic therapies. J Am Acad Dermatol. (2020) 82:1445–86. 10.1016/j.jaad.2020.02.044 32119894

[16] Choi JH Lee DH Song WJ Choi M Kwon JW Kim GW The KAAACI/KDA evidence-based practice guidelines for chronic spontaneous urticaria in Korean adults and children: part 2. Management of H1-antihistamine-refractory chronic urticaria. Allergy Asthma Immunol Res. (2020) 12:750–70. 10.4168/aair.2020.12.5.750 32638557 PMC7346997

[17] Bishnoi A Parsad D Vinay K Kumaran MS. Phototherapy using narrowband ultraviolet B and psoralen plus ultraviolet A is beneficial in steroid-dependent antihistamine-refractory chronic urticaria: a randomized, prospective observer-blinded comparative study. Br J Dermatol. (2017) 176:62–70. 10.1111/bjd.14778 27258736

[18] Muntean IA Pintea I Bocsan IC Dobrican CT Deleanu D. COVID-19 disease leading to chronic spontaneous urticaria exacerbation: a Romanian retrospective study. Healthcare. (2021) 9:1144. 10.3390/healthcare9091144 34574918 PMC8470475

[19] Chen J Zeng X Chen Q Liang B Peng L Li H Efficacy of NB-UVB as add-on therapy to antihistamine in the treatment of chronic urticaria: a systematic review and meta-analysis. Dermatol Ther. (2021) 11:681–94. 10.1007/s13555-021-00510-2 PMC8163931 33738748

[20] Torres AE Lyons AB Hamzavi IH Lim HW. Role of phototherapy in the era of biologics. J Am Acad Dermatol. (2021) 84:479–85. 10.1016/j.jaad.2020.04.095 32339702 PMC7194984

[21] Kurz B Berneburg M Bäumler W Karrer S. Phototherapy: theory and practice. J Dtsch Dermatol Ges. (2023) 21:882–97. 10.1111/ddg.15126 37485907

[22] Barros NdM Sbroglio LL Buffara MdO Baka JeS Pessoa AdS Azulay-Abulafia L. Phototherapy. An Bras Dermatol. (2021) 96:397–407. 10.1016/j.abd.2021.03.001 33849754 PMC8245715

[23] Vieyra-Garcia PA Wolf P. A deep dive into UV-based phototherapy. Pharmacol Ther. (2021) 222:107784. 10.1016/j.pharmthera.2020.107784 33316286

[24] Hong JY Kim MH Lee JH Han HS Seo SJ Park KY Phototherapy may be a useful adjuvant therapy for retractable chronic spontaneous urticaria: a systematic review. Photochem Photobiol. (2020) 96:738–40. 10.1111/php.13260 32144786

[25] Sheikh G Latif I Lone KS Hassan I Jabeen Y Keen A. Role of adjuvant narrow band ultraviolet B phototherapy in the treatment of chronic urticaria. Indian J Dermatol. (2019) 64:250. 10.4103/ijd.IJD_475_16 31148870 PMC6537687

[26] Khafagy NH Salem SAM Ghaly EG. Comparative study of systemic psoralen and ultraviolet A and narrowband ultraviolet B in treatment of chronic urticaria. Photodermatol Photoimmunol Photomed. (2013) 29:12–7. 10.1111/phpp.12008 23281692

[27] Hannuksela M Kokkonen EL. Ultraviolet light therapy in chronic urticaria. Acta Derm Venereol. (1985) 65:449–50. 10.2340/0001555565449450 2416176

[28] Olafsson JH Larkö O Roupe G Granerus G Bengtsson U. Treatment of chronic urticaria with PUVA or UVA plus placebo: a double-blind study. Arch Dermatol Res. (1986) 278:228–31. 10.1007/BF00412929 2425755

[29] Engin B Ozdemir M Balevi A Mevlitoğlu I. Treatment of chronic urticaria with narrowband ultraviolet B phototherapy: a randomized controlled trial. Acta Derm Venereol. (2008) 88:247–51. 10.2340/00015555-0434 18480923

[30] Aydogan K Karadogan SK Tunali S Saricaoglu H. Narrowband ultraviolet B (311 nm, TL01) phototherapy in chronic ordinary urticaria. Int J Dermatol. (2012) 51:98–103. 10.1111/j.1365-4632.2011.05056.x 22182386

[31] Berroeta L Clark C Ibbotson SH Ferguson J Dawe RS. Narrow-band (TL-01) ultraviolet B phototherapy for chronic urticaria. Clin Exp Dermatol. (2004) 29:97–8. 10.1111/j.1365-2230.2004.01442.x 14723739

[32] Midelfart K Moseng D Kavli G Stenvold SE Volden G. A case of chronic urticaria and vitiligo, associated with thyroiditis, treated with PUVA. Dermatology. (1983) 167:39–41. 10.1159/000249743 6628797

